# 803. The Impact of Bundled Interventions to Decrease Transmission of Drug-Resistant *Pseudomonas aeruginosa* from Wastewater Drain Sites on a Hematologic Malignancy/Hematopoietic Stem Cell Transplant Unit

**DOI:** 10.1093/ofid/ofab466.999

**Published:** 2021-12-04

**Authors:** Lauren Fontana, Morgan Hakki, Richard Zhang, William Messer, Grace Walker-Stevenson, Amy Laird, Lynne Strasfeld

**Affiliations:** 1 University of Minnesota, Minneapolis, Minnesota; 2 Oregon Health and Science University, Portland, OR; 3 University of Oregon, Corvallis, Oregon; 4 Oregon Health and Science University-Portland State University, Portland, Oregon; 5 Oregon Health & Science University, Portland, Oregon

## Abstract

**Background:**

Wastewater drain (WWD) sites are an important reservoir for amplification, propagation and transmission of multidrug resistant organisms. We observed an increase in the incidence of carbapenem and fluoroquinolone non-susceptible (CP-NS and FQ-NS) *P. aeruginosa* bloodstream infections (BSI) among patients on our hematologic malignancies (HM) and hematopoietic cell transplant (HCT) unit. The incidence of CP-NS/FQ-NS *P. aeruginosa* BSI from 2012 through May 2021 is represented in Figure 1. We sought to determine the impact of low-cost, low-barrier interventions targeting WWD sites on the prevalence of patient and environmental *P. aeruginosa* colonization and incidence of BSI.

Figure 1. Incidence of P. aeruginosa BSI, 2012 through May 2021

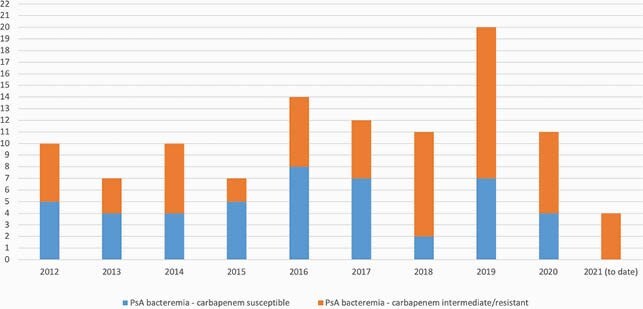

**Methods:**

Behavioral and structural interventions to limit acquisition from WWD sites were informed by an environmental analysis and rolled out in staged fashion beginning in September 2019. Pre- and post-intervention colonization surveys were performed on the unit to assess for patient and WWD site *P. aeruginosa* colonization. Whole genome sequencing (WGS) was performed on select isolates. A sensitivity analysis performed accounted for the unconfirmed patient isolates. BSI data was collected retrospectively.

**Results:**

Characteristics of the pre- and post-intervention groups are presented in Table 1. Five of 27 (18.5%) and 1 of 26 (3.8%) patients in the pre- and post-intervention point prevalence survey, respectively, were confirmed to be colonized with *P. aeruginosa* (Figure 2), corresponding to a prevalence rate ratio of 0.21 (0.03,1.66). If the two indeterminate samples in the pre-intervention period were positive, the prevalence rate ratio would instead be 0.15 (0.02,1.12). The most frequent *P. aeruginosa* strains identified by WGS from the patients and environment were 111, 308 and 446. At least 87% of rooms were colonized with *P. aeruginosa* from at least one WWD site, from pre- and post-intervention periods (Table 2). Table 1. Demographic and clinical characteristics of patients in each epoch. Results are given as percent (frequency) unless otherwise noted. Chi square test was used unless otherwise noted.

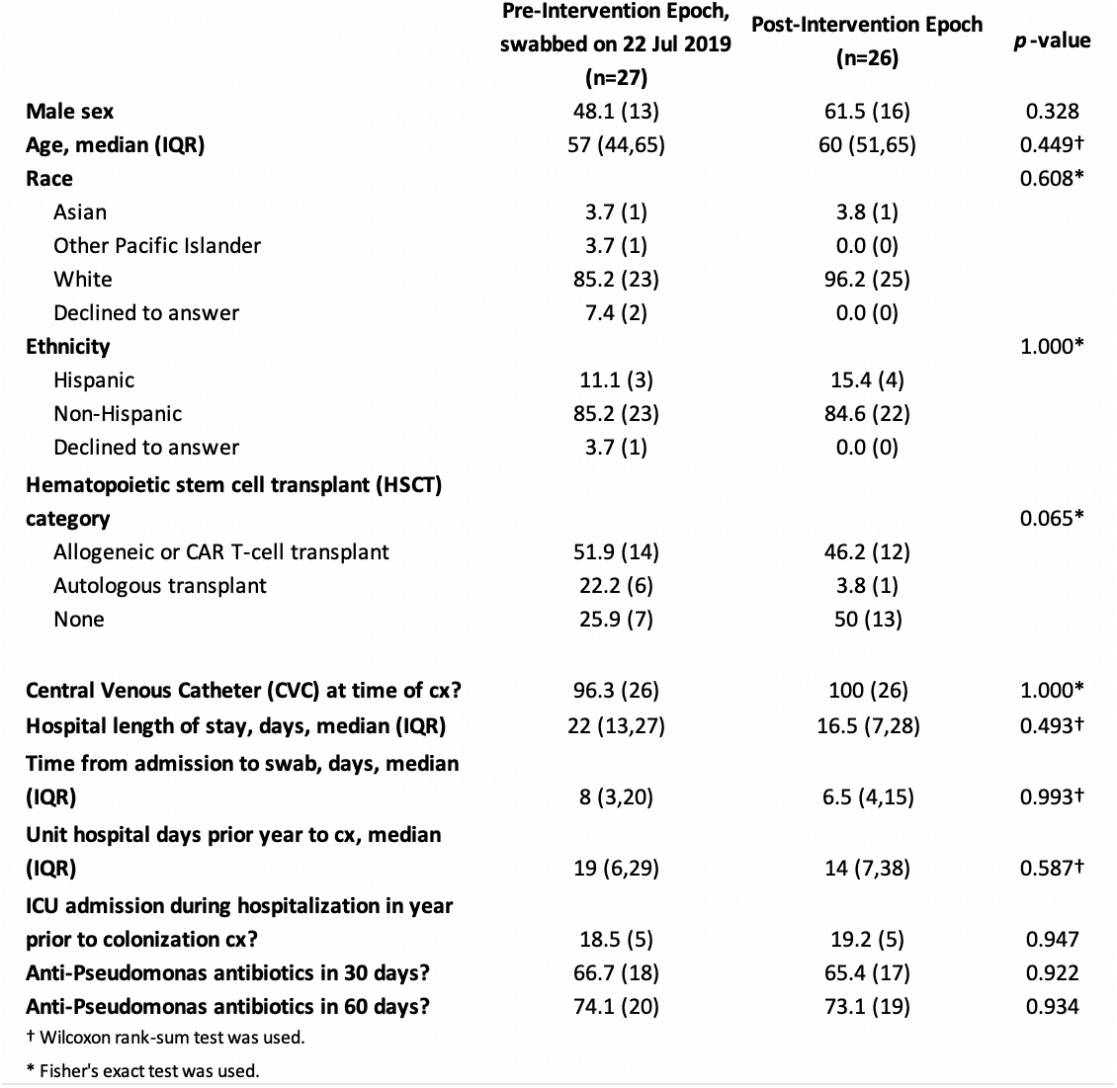

Figure 2. Proportion of patients colonized with P. aeruginosa Positive: Colonized with P. aeruginosa, confirmed by WGS; Unknown: Phenotype of isolate suggestive of P. aeruginosa, WGS not performed; Negative: No growth on selective agar or non-P aeruginosa identification on WGS

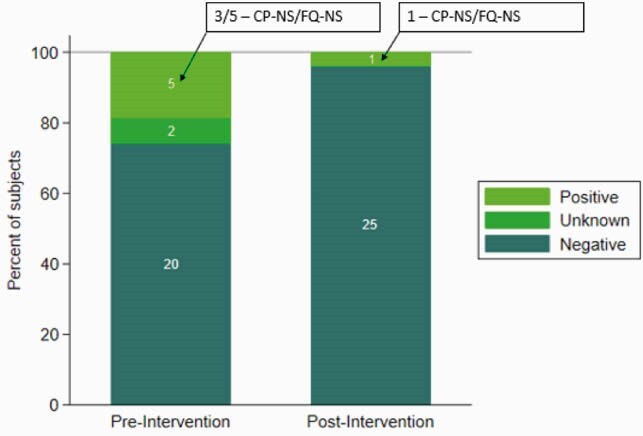

Table 2. WWD site colonization, by phenotypic and WGS determination. Fisher’s exact test was used unless otherwise noted.

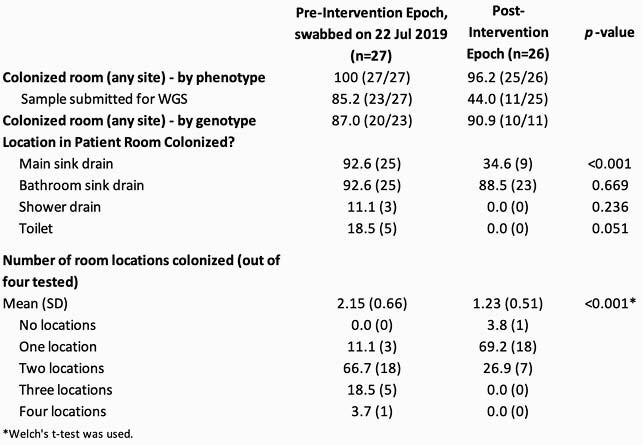

**Conclusion:**

*P. aeruginosa* WWD colonization on our HM/HCT unit may predispose patients to colonization and BSI. The prevalence of patient colonization decreased following implementation of the interventions, despite persistent environmental colonization. We will follow the incidence of *P. aeruginosa* BSI to determine the long-term impact of these interventions.

**Disclosures:**

**All Authors**: No reported disclosures

